# Molecular Mechanisms Underlying the Progression of Aortic Valve Stenosis: Bioinformatic Analysis of Signal Pathways and Hub Genes

**DOI:** 10.3390/ijms24097964

**Published:** 2023-04-27

**Authors:** Taiki Tojo, Minako Yamaoka-Tojo

**Affiliations:** 1 Department of Cardiovascular Medicine, Kitasato University School of Medicine, Sagamihara 252-0374, Japan; 2 Department of Rehabilitation, Kitasato University School of Allied Health Sciences, Sagamihara 252-0373, Japan

**Keywords:** expression profiling, adhesion molecules, inflammation, endothelial cells, signaling

## Abstract

The calcification of the aortic valve causes increased leaflet stiffness and leads to the development and progression of stenotic aortic valve disease. However, the molecular and cellular mechanisms underlying stenotic calcification remain poorly understood. Herein, we examined the gene expression associated with valve calcification and the progression of calcific aortic valve stenosis. We downloaded two publicly available gene expression profiles (GSE83453 and GSE51472) from NCBI-Gene Expression Omnibus database for the combined analysis of samples from human aortic stenosis and normal aortic valve tissue. After identifying the differentially expressed genes (DEGs) using the GEO2R online tool, we performed Gene Ontology and Kyoto Encyclopedia of Genes and Genomes pathway enrichment analyses. We also analyzed the protein–protein interactions (PPIs) of the DEGs using the NetworkAnalyst online tool. We identified 4603 upregulated and 6272 downregulated DEGs, which were enriched in the positive regulation of cell adhesion, leukocyte-mediated immunity, response to hormones, cytokine signaling in the immune system, lymphocyte activation, and growth hormone receptor signaling. PPI network analysis identified 10 hub genes: *VCAM1*, *FHL2*, *RUNX1*, *TNFSF10*, *PLAU*, *SPOCK1*, *CD74*, *SIPA1L2*, *TRIB1*, and *CXCL12*. Through bioinformatic analysis, we identified potential biomarkers and therapeutic targets for aortic stenosis, providing a theoretical basis for future studies.

## 1. Introduction

Severe aortic valve stenosis (AS) is a common valvular heart disease that affects millions of individuals worldwide, particularly those aged 75 years and above [1]. It is characterized by a narrowing of the aortic valve orifice, resulting in the obstruction of blood flow from the left ventricle to the aorta [2]. The pathogenesis of AS involves an interplay of genetic and environmental factors leading to the progressive fibrocalcific degeneration of the aortic valve leaflets [3,4]. Inflammatory cells, including macrophages, leukocytes, dendritic cells, mast cells, and platelets, have been identified in stenotic aortic valve specimens [5,6]. Osteogenically differentiated valve endothelial cells secrete adhesion factors and participate in immunomodulatory functions [7].

Recent advances in transcatheter aortic valve implantation (TAVI) and surgical aortic valve replacement (SAVR) have improved the prognosis of patients with severe AS. Although the optimal timing of intervention and choice of valve replacement strategy remain contentious issues [8], the timing currently depends mostly on clinical data, especially the development of symptoms and echocardiographic changes in the left ventricle. However, this determination of timing is notoriously difficult in patients with comorbid conditions, such as chronic obstructive pulmonary disease, which could result in similar symptoms. The choice of valve replacement, whether it be biological versus mechanical or SAVR versus TAVI, depends on the patient’s age, comorbidities, and life expectancy. However, there is a need to better understand the molecular pathogenesis of AS in order to develop personalized treatment strategies.

Array-based expression profiling is a powerful technique that allows researchers to measure the activity of thousands of genes in a single experiment [9]. By analyzing the gene expression patterns in diseased tissue samples, researchers can gain insight into the molecular mechanisms underlying the disease and identify potential therapeutic targets [10,11]. A large number of microarray data are freely available from the open access NCBI-Gene Expression Omnibus (GEO) database. In this study, we searched and screened the transcriptome microarray data of aortic stenosis from the GEO Expression Omnibus (GSE) series studies and used bioinformatic analysis to identify differentially expressed genes (DEGs) between patients with AS and controls. Gene Ontology (GO) and Kyoto Encyclopedia of Genes and Genomes (KEGG) pathway enrichment analyses were performed to detect statistically significant signaling pathways in the DEGs. A protein–protein interaction (PPI) network was also established to identify hub genes. As shown in Table 1, several relevant and novel techniques exist for quantitative analysis using the genes differentially expressed in a disease condition or disease-related exposure [12].

In this study, we aimed to identify the genetic factors and pathways that contribute to the development and progression of severe AS, with a focus on aortic valve tissue. We performed a comprehensive genetic profiling of aortic valve tissue from patients with severe AS undergoing TAVI or SAVR and compared the results with those obtained with tissue samples from control subjects. Our findings provide insight into the mechanisms underlying AS, a direction for the discovery of diagnostic biomarkers and targeted therapeutic strategies for AS, and guide the development of new therapeutic strategies for this prevalent valvular heart disease.

## 2. Results

### 2.1. Microarray Data Information

**Figure 1:**
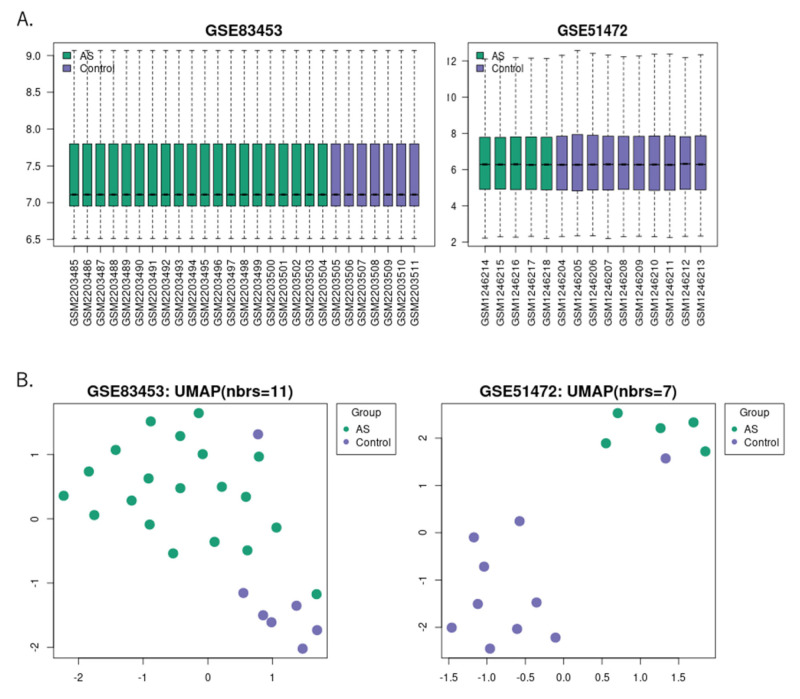
Gene expression and UMAP for each sample: (**A**) Gene expression data from GSE83453 and GSE51472, with the sample list on the horizontal axis and the log2-converted gene expression values on the vertical axis. Green bars represent tissue samples from patients with aortic stenosis, and purple bars represent control samples. (**B**) UMAP analysis was performed on each sample from the GSE83453 and GSE51472 datasets. Data from aortic stenosis and control subjects were relatively distinct, with green dots representing tissue samples from patients with aortic stenosis and purple dots representing control samples. UMAP, uniform manifold approximation and projection.

We selected two gene expression profiles, GSE83453 and GSE51472, to identify the DEGs in aortic stenosis patients compared with those in healthy individuals. Raw data were downloaded and processed using the Affy package. The boxplots of the data, shown in Figure 1A, allowed us to perform subsequent genetic difference analyses. We utilized uniform manifold approximation and projection (UMAP), a dimension reduction technique, to visualize how the samples were related to each other. The UMAP results showed that the data for the aortic stenosis and control groups were relatively distinguishable, as shown in Figure 1B.

### 2.2. DEG Analysis in AS

**Figure 2:**
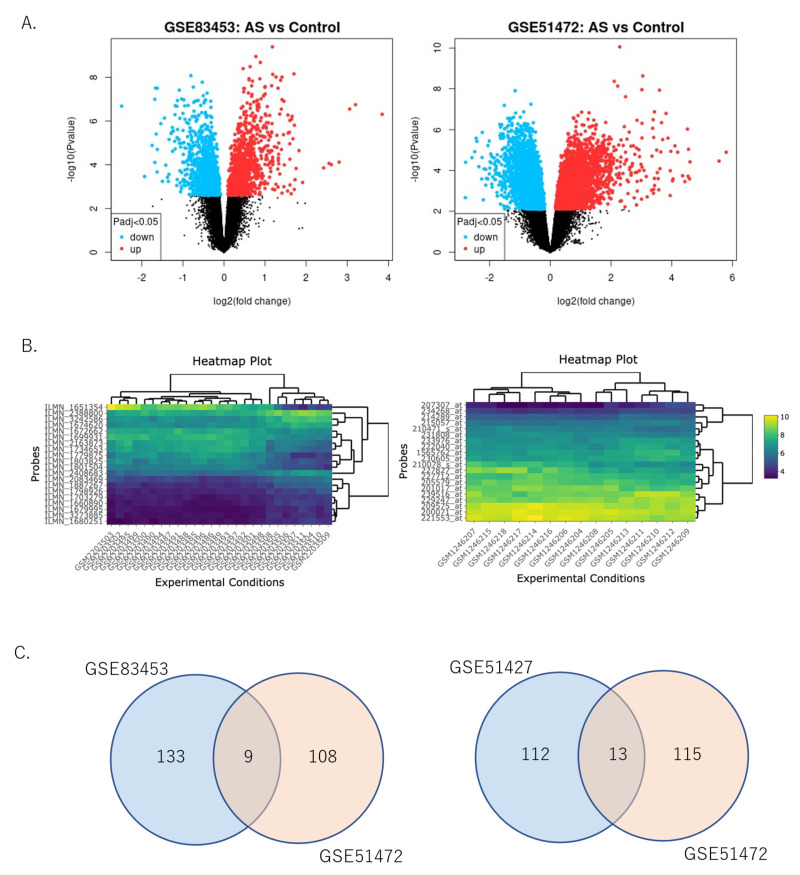
Volcano plots, cluster heatmaps, and DEG Venn diagrams for the GSE83453 and GSE51427 datasets: (**A**) Volcano plots for the GSE83453 (**left panel**) and GSE51427 (**right panel**) datasets. Red dots represent upregulated genes based on an adjusted *p*-value < 0.05 and log2FC ≥ 1. Blue dots represent downregulated genes based on an adjusted *p*-value < 0.05 and log2FC ≥ −1. Black dots represent genes with no significant difference. (**B**) Cluster heatmap of the top 20 significant genes of the GSE83453 (**left**) and GSE51427 (**right**) datasets. Yellow indicates upregulated gene expression, blue indicates downregulated gene expression, and green indicates no significant change in gene expression. (**C**) Venn diagram of DEGs in the highly expressed upregulated or downregulated top 300 genes in each dataset (GSE83453 and GSE51427): (**left panel**) five overlapping DEGs upregulated in the two datasets; (**right panel**) eight overlapping DEGs downregulated in the two datasets. GSE83453 had 258 DEGs, comprising 138 upregulated and 120 downregulated genes, while GSE51427 included 236 DEGs, comprising 113 upregulated and 123 downregulated genes. DEG, differentially expressed gene; AS, aortic valve stenosis; log2FC; log2 fold change.

The DEGs between AS and control tissue identified from GSE83453 and GSE51472 were analyzed using the “limma” package. Differential gene expression analysis was performed for 10,875 genes, 4603 of which were upregulated and 6272 were downregulated. Of these, 1127 genes were upregulated and 1255 were downregulated in GSE83453 (Figure 2A, left), whereas 3476 genes were upregulated and 5017 were downregulated in GSE51472 (Figure 2A, right).

The top 20 genes in GSE83453 (Figure 2B, left) and GSE51472 (Figure 2B, right) are shown in the heatmap. Among the significantly highly expressed top 100 genes extracted from the top 300 upregulated or downregulated genes in each dataset (GSE83453 and GSE51472), GSE83453 had 267 DEGs, comprising 142 upregulated and 125 downregulated genes, and GSE51427 had 245 DEGs, comprising 117 upregulated and 128 downregulated genes. A Venn diagram was constructed to obtain the final set of the DEGs (Figure 2C). Finally, 22 DEGs were identified, comprising 9 upregulated and 13 downregulated genes (Figure 2C). Table 2 shows the upregulated and downregulated DEGs from the top 100 genes sorted by the average log2 fold change (log2FC) value for the two datasets.

**Figure 3:**
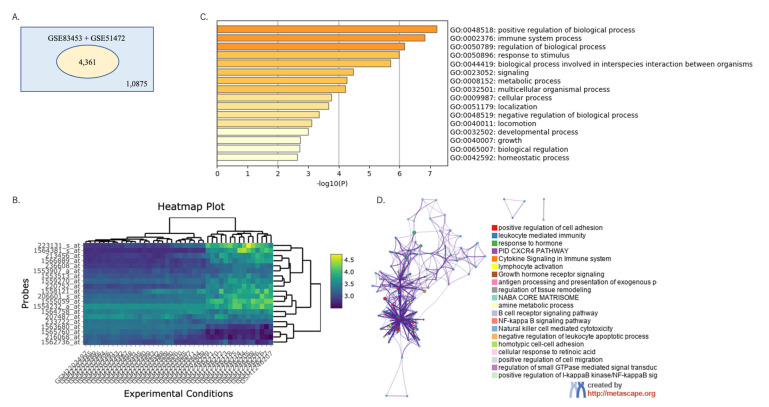
Differential gene expression analysis and functional enrichment analysis of DEGs using Metascape: (**A**) Venn diagram plot of differential gene expression analysis using GEOexplorer. A total of 10,875 genes were identified from a combined dataset of GSE83453 and GSE51472. Among these genes, 4361 genes were identified as common DEGs. (**B**) Heatmap plot generated by GEOexplorer using the combined GSE83453 and GSE51472 dataset. The top 20 most significant genes were analyzed. (**C**) Bar graph of enriched terms across the top 100 genes list from the GSE83453 and GSE51472 datasets, colored according to *p*-values. (**D**) Network of the top 20 clusters of enriched terms in Metascape. Cluster identification is represented by color, and the similarity score is represented by the thickness of the edge. DEGs, differentially expressed genes.

Among the combined data of GSE83453 and GSE51472, which included 10,875 genes for both analyses, the top DEGs were analyzed using GEOexplorer (Table 3). Among them, 4361 genes were identified as common DEGs (Figure 3A). The top 20 most significant genes were analyzed using a heatmap plot generated with GEOexplorer using the combined GSE83453 and GSE51472 datasets (Figure 3B).

### 2.3. Functional Enrichment Analysis

Functional enrichment analysis was performed using Metascape to annotate and integrate the GO and KEGG pathway enrichment analyses, and the results are presented in Figure 3C,D. We identified statistically enriched terms and calculated cumulative hypergeometric *p*-values and enrichment factors. KEGG pathway enrichment analysis revealed important pathways, including the positive regulation of cell adhesion, leukocyte-mediated immunity, response to hormones, cytokine signaling in the immune system, lymphocyte activation, growth hormone receptor signaling, antigen processing, and the presentation of exogenous peptide antigen via major histocompatibility complex class II, the regulation of tissue remodeling, amine metabolic process, B-cell receptor signaling pathway, and NF-kB signaling pathway (Figure 3C). The relationships between the enriched terms are illustrated in a 3D network of the top 20 clusters of enriched terms in Metascape, where cluster identification is represented by color, and the similarity score is represented by edge thickness (Figure 3D). The analysis showed that these DEGs were mainly enriched in the positive regulation of cell adhesion, leukocyte-mediated immunity, response to hormones, cytokine signaling in the immune system, lymphocyte activation, and growth hormone receptor signaling.

### 2.4. PPI Network Analysis and Hub Gene Identification

**Figure 4:**
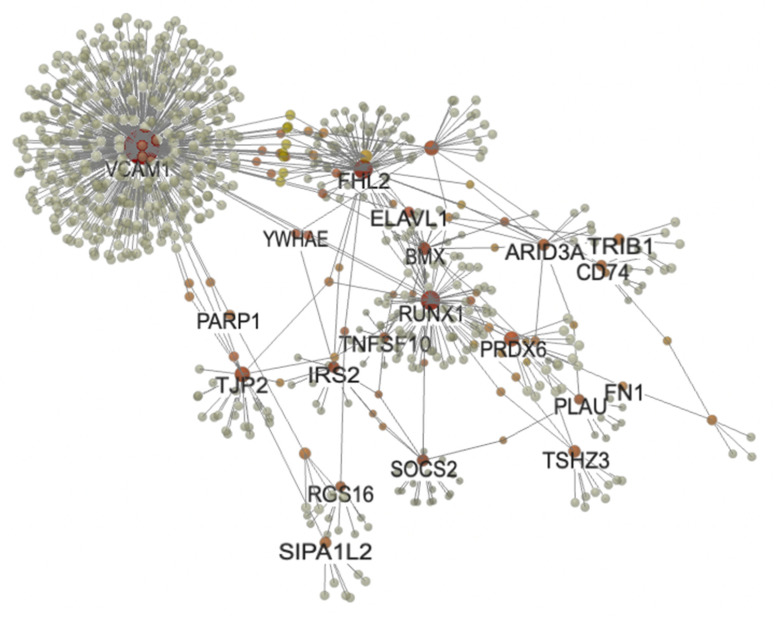
PPI network analysis of DEGs in patients with aortic sclerosis datasets (GSE83453 and GSE51472). The 3D-PPI network analysis was performed using NetworkAnalyst, with DEGs highlighted in orange. The top 10 hub genes with the highest number of connections are listed (Table 4). PPIs, protein–protein interactions; DEGs, differently expressed genes; VCAM1, vascular cell adhesion molecule 1; FHL2, four-and-a-half LIM domain protein 2; RUNX1, runt-related transcription factor 1; TNFSF10, TNF superfamily member 10; PLAU, plasminogen activator, urokinase receptor; SPOCK1, sparc/osteonectin, cwcv, and kazal-like domains proteoglycan (testican) 1; CD74, cluster of differentiation 74; SIPA1L2, signal-induced proliferation-associated 1 like 2; TRIB1, Tribbles pseudokinase 1; CXCL12, C-X-C motif chemokine ligand 12; YWHA, tyrosine 3-monooxygenase/tryptophan 5-monooxygenase activation protein zeta; PARP1, poly(ADR-Ribose) polymerase 1; TJP, tight junction protein; IRS2, insulin receptor substrate; BMX, BMX non-receptor tyrosine kinase; SOCS2, suppressor of cytokine signaling 2; PRDX6, peroxiredoxin 6; ARID3A, AT-rich interaction domain 3; TSHZ3, teashirt zinc finger homeobox 3; RGS16, regulator of G-protein signaling 16; ELAVL1, ELAV-like protein 1; FN1, fibronectin 1.

We performed a PPI network analysis of DEGs in patients with aortic stenosis using the NetworkAnalyst tool. To construct a tissue-specific network, we used the DifferentialNet database and selected the top 52 known genes from the top 100 DEGs identified in the combined GSE83453 and GSE51472 datasets. We identified the hub genes for aortic stenosis by choosing a minimal connectivity network. The resulting 3D-PPI network consisted of 786 nodes and 842 edges (Figure 4). Based on their connectivity in the PPI network, we evaluated the top 10 hub genes: *VCAM1*, *FHL2*, *RUNX1*, *TNFSF10*, *PLAU*, *SPOCK1*, *CD74*, *SIPA1L2*, *TRIB1*, and *CXCL12* (Table 4). All the identified hub genes were upregulated in patients with aortic stenosis.

## 3. Discussion

Our analysis of DEGs in patients with AS and healthy individuals sheds light on the mechanisms underlying this disease. Overall, our findings suggest that the pathways involved in the positive regulation of cell adhesion, leukocyte-mediated immunity, response to hormones, and cytokine signaling in the immune system may be implicated. Additionally, the identification of ten hub genes, namely *VCAM1*, *FHL2*, *RUNX1*, *TNFSF10*, *PLAU*, *SPOCK1*, *CD74*, *SIPA1L2*, *TRIB1*, and *CXCL12*, provides potential targets for future therapeutic interventions in AS.

In this study, we obtained four microarray datasets (GSE83453, GSE51472, GSE115119, and GSE77287) from the Gene Expression Omnibus (GEO) database and conducted individual bioinformatic analyses to identify DEGs. After data screening, we selected two datasets (GSE83453 and GSE51472) because the remaining two did not show any significant differences between the AS and control samples. The top 300 upregulated or downregulated genes from each selected dataset were separately extracted and intersected using the Venn diagram web tool to obtain a combined set of 490 integrated DEGs, including 250 upregulated and 240 downregulated genes. Although we conducted GEO2R analyses and identified 7 upregulated and 13 downregulated genes with significantly high expression, we could not identify any significant PPI network or hub genes among these hits. Thus, to identify more prominent factors, we integrated all the DEGs from both datasets (GSE83453 and GSE51472) and subjected them to GO and KEGG pathway enrichment analyses as well as tissue-specific PPI network analysis.

Inflammation plays a crucial role in the occurrence and progression of AS and calcification [7]. A previous study has reported the presence of inflammation in 95% of calcified aortic valve tissues [13]. Moreover, leukocyte density has been found to be associated with the progression of AS, with inflammatory cells observed around calcific nodules and areas of angiogenesis [14]. Additionally, the platelet-to-lymphocyte ratio is associated with the progression of AS [15]. Gene expression patterns in the tissue samples obtained from the aortic valves of patients with severe aortic stenosis have been compared with samples from healthy controls in previous studies [11,16]. However, one major difference between our study and previous reports is our focus on clinical-practice-oriented grouping. Specifically, the aortic valve specimens from patients with AS who require invasive surgery were defined as “AS”, while the specimens from patients with calcified aortic valves without stenosis were included in the control group. Consequently, a different set of hub genes was identified compared with that from previous studies [7,13,17,18].

In the present study, the differentially expressed gene vascular cell adhesion molecule (VCAM1) was found to be highly expressed in the stenotic aortic valves of patients with AS. A previous study has shown that VCAM1 is highly expressed in the aortic valves of diabetic/atherosclerotic ApoE-deficient mice and is considered a potential target for nanocarriers developed to block the progression of AS [19]. VCAM1 is a transmembrane sialoglycoprotein that is often used for targeted drug delivery to endothelial cells due to its inducible expression on the cell surface in pathological conditions [20,21,22,23]. Moreover, there is an increased expression of VCAM1 in aortic valve interstitial cells after exposure to IFN-γ-and lipopolysaccharide [24]. Efficient drug delivery systems for AS using nanomedicine are currently being examined, with a particular focus on VCAM1-targeted PEGylated lipopolyplexes as a potential encapsulating drug delivery system [23]. However, translating this technology into clinical practice is difficult and may have serious limitations.

After VCAM1, the next most highly expressed gene was four-and-a-half LIM domain protein 2 (FHL2). FHL2 is believed to act as a specific adaptor protein that couples metabolic enzymes to sites of high energy consumption in the sarcomeres by interacting with titin/connectin [25]. FHL2 is highly expressed in vascular cells, including endothelial and smooth muscle cells, where it functions as a scaffolding protein that modulates the signal transduction pathways crucial for the vasculature [26,27]. Several LIM domain proteins play a role in the development and maintenance of cellular architecture by adhering to the myofibrils of the cytoskeleton. Altered FHL2 expression in heart failure is associated with the disruption of the normal subcellular localization of phosphofructokinase 2, adenylate kinase, and creatine kinase M isoform, as well as the reduced activity of phosphofructokinase 2 and adenylate kinase [28]. In mouse aortic endothelial cells exposed to PM2.5, autophagy-induced FHL2 upregulation promotes IL-6 production via the activation of the NF-kB pathway [29].

Additionally, runt-related transcription factor 1 (RUNX1) was identified as a highly expressed gene in the stenotic aortic valves of patients with AS. RUNX1 is a transcription factor that plays a crucial role in cardiovascular development and homeostasis [30,31]. It regulates the expression of the genes involved in cell proliferation, differentiation, and apoptosis and has been shown to be involved in the pathogenesis of cardiovascular disease [32,33]. Furthermore, a previous study reported that RUNX1 is involved in the development of calcific aortic valve disease and is upregulated in human aortic valve interstitial cells treated with calcifying media [34]. The overexpression of RUNX1 has been shown to promote calcification and osteogenic differentiation of aortic valve interstitial cells through the BMP2 and Wnt/beta-catenin signaling pathways [35]. RUNX1 interacts with NOTCH1, a key regulator of cardiovascular development and calcification, to regulate valve calcification [36]. Valvular calcification is the result of an active process involving endothelial dysfunction, subendothelial lipid (oxidized LDL) deposition, inflammation, and bone formation (expression of osteocalcin, osteonectin, and osteopontin). Polymorphisms, such as mutations in the gene encoding the vitamin D receptor, or those affecting the synthesis of apolipoprotein or the transcription factor NOTCH 1, have been shown to be involved.

Network analysis is a powerful approach that can aid in the identification of hub genes and the discovery of potential therapeutic targets [37]. In the present study, we constructed a PPI network of DEGs and identified 10 hub genes based on their degrees of association.

## 4. Materials and Methods

### 4.1. Microarray Data Source and Screening

GEO is a public database that contains microarray, next-generation sequencing, and other high-throughput sequencing data (https://www.ncbi.nlm.nih.gov/, accessed on 22 April 2023). We used the search terms “aortic valve stenosis” and “microarray” and identified four transcriptomic datasets after filtering by “Homo sapiens” and “expression profiling by array”: GSE83453, GSE51472, GSE115119, and GSE77287. Next, we selected human transcriptome datasets that utilized the same GEO platform and analyzed aortic valve tissue; the resulting datasets were GSE83453 and GSE51472. These data showed significant differences between stenotic and control aortic valves. The GSE83453 dataset was based on the GPL10558 platform (Illumina HumanHT-12 V4.0, expression bead chip), whereas the GSE51472 dataset was based on the GPL570 platform (Affymetrix Human Genome U133 Plus 2.0 Array). The survey information is presented in Table 5.

GSE83453 [8] contains 7 normal valve tissue samples and 20 stenotic aortic valve tissue samples (10 bicuspid aortic valves and 10 tricuspid aortic valves) from Canada, while GSE51472 [9] contains 10 control aortic samples (5 normal aortic valves and 5 calcified aortic valves without aortic stenosis) and 5 aortic stenosis samples from Finland. All aortic samples were obtained from patients with AS undergoing aortic replacement surgery. Normal control valves were obtained from heart transplant patients or from patients with ascending aortic disease who required aortic valve replacement [39].

### 4.2. Screening and Integration of DEGs

The “limma” package was used for DEG analysis [14]. Gene expression values were log2-transformed and normalized after modified range migration algorithm processing and fitted to a linear model using the weighted least squares method. Differential expression analysis was performed between the data from patients with aortic stenosis and controls. The “limma” package calculated log2FC and the false discovery rate (FDR)-adjusted values for each gene. The FDR corrects values using the Benjamini–Hochberg method to control the number of false positives for multiple tests. Adjusted values < 0.05 and |log2FC| ≥ 1.0 were considered significant DEGs. Due to a significant batch effect between the GSE83453 and GSE51472 datasets, the DEGs for each dataset were analyzed and obtained separately. The final list of DEGs was generated by integrating the two datasets using the Venn diagram tool (https://bioinformatics.psb.ugent.be/webtools/Venn accessed on 22 April 2023).

### 4.3. Differential Gene Expression Analysis

Differential gene expression analyses were performed using the combined data from the two datasets (GSE83453 and GSE51472) through Venn diagram plot analysis and heatmap plot visualization using online GEOexplorer (https://geoexplorer.rosalind.kcl.ac.uk accessed on 22 April 2023).

### 4.4. Pathway Enrichment Analysis

We performed GO and KEGG pathway enrichment analyses using the “ClusterProfiler” and “org.Hs.eg.db” packages with adjusted *p*-value cutoffs set to 0.05. To visualize the relationships between the enriched terms, Metascape (https://metascape.org/ accessed on 22 April 2023) was used to annotate and integrate the GO and KEGG analysis results. The adjusted *p*-value cutoff for GSEA was set to <0.05, and the Benjamini–Hochberg method was used to adjust the raw values. Additionally, we performed the KEGG pathway enrichment analysis of integrated DEGs using the DAVID webtool (https://david.ncifcrf.gov/ accessed on 22 April 2023), with statistical significance set at *p* < 0.05. To visualize the relationships between the enriched terms, we used Metascape (https://metascape.org/ accessed on 22 April 2023) to annotate and integrate the GO and KEGG analyses.

### 4.5. Tissue-Specific PPI Network Analysis and Hub Gene Identification

We performed aortic tissue-specific PPI network analysis using various gene lists and NetworkAnalyst [16], an online bioinformatic tool with powerful features and an easy-to-use interface (https://www.networkanalyst.ca/ accessed on 22 April 2023). The PPI data for human tissues (aorta and coronary arteries) were obtained from the DifferentialNet database (https://netbio.bgu.ac.il/diffnet/ accessed on 22 April 2023) [18]. Hub genes were identified through selected minimal connectivity networks, where each node represents a protein encoded by a gene, and the connections between nodes represent protein interactions. The nodes with the greatest connectivity were considered core proteins or hub genes with essential biological regulatory functions in aortic stenosis. However, further validation studies are required to explore the therapeutic potential of these targets in this patient group.

## 5. Conclusions

In conclusion, our analysis of DEGs in patients with AS and healthy individuals suggest pathways involved in the positive regulation of cell adhesion, leukocyte-mediated immunity, hormone responses, and cytokine signaling in the immune system may be implicated in the mechanisms underlying disease. Additionally, the identification of ten hub genes, including *VCAM1*, *FHL2*, *RUNX1*, *TNFSF10*, *PLAU*, *SPOCK1*, *CD74*, *SIPA1L2*, *TRIB1*, and *CXCL12*, may provide potential targets for future therapeutic interventions in aortic stenosis.

## Figures and Tables

**Table 1:** Overview of the informatic pipeline to analyze various omic datasets generated from biological experiments.

Identification of Differentially Expressed Genes			
Bioinformatic data warehousing Public annotations and datasets + Combined datasets			
Regulatory network analysis	Data integration/Pathway mapping	Machine-learning-based methods for marker identification	Deep learning methods
Multiomic data analysis			

**Table 2:** DEGs from the top 100 genes from each dataset (GSE83453 and GSE51472), sorted by the average log2FC size. DEGs, differentially expressed genes; log2FC, log2 fold change.

DEGs	Gene Title (*Gene Symbol*)
Upregulated	secreted phosphoprotein 1 (*SPP1*), matrix metallopeptidase 9 (*MMP9*), joining chain of multimeric IgA and IgM (*JCHAIN*), stathmin 2 (*STMN2*), matrix metallopeptidase 12 (*MMP12*), CD52 molecule (*CD52*), Fc fragment of IgG receptor 1B (*FCGR1B*), lysozyme (*LYZ*), hematopoietic cell signal transducer (*HCST*)
Downregulated	collagen type VI alpha 6 chain (*COL6A6*), immunoglobulin superfamily member 10 (*IGSF10*), ATPase Na+/K+ transporting subunit alpha 2 (*ATP1A2*), angiopoietin like 7 (*ANGPTL7*), microtubule associated protein tau (*MAPT*)m neuron specific gene family member 1 (*NSG1*), immunoglobulin superfamily member 10 (*IGSF10*), HAND2 antisense RNA 1 (*HAND2-AS1*), complement C6 (*C6*), plasmolipin (*PLLP*), sclerostin (*SOST*), lamin subunit gamma 3 (*LAMC3*), solute carrier family 6 member 4 (*SLC6A4*)

**Table 3:** Top 30 differentially highly expressed genes.

Gene	ID	David Gene Name
1564381_S_AT	100505876	CEBPZ opposite strand (*CEBPZOS*)
1558121_AT	80034	cysteine and serine rich nuclear protein 3 (*CSRNP3*)
227462_AT	64167	endoplasmic reticulum aminopeptidase 2 (*ERAP2*)
236608_AT	165082	adhesion G protein-coupled receptor F3 (*ADGRF3*)
1561094_A_AT	387601	solute carrier family 22 member 25 (*SLC22A25*)
224224_S_AT	50940	phosphodiesterase 11A (*PDE11A*)
1564758_AT	26074	cilia and flagella associated protein 61 (*CFAP61*)
219850_S_AT	26298	ETS homologous factor (*EHF*)
236597_AT	133688	UDP glycosyltransferase family 3 member A1 (*UGT3A1*)
220752_AT	51145	PBX3 divergent transcript (*PBX3-DT*)
1566889_AT	63892	THADA armadillo repeat containing (*THADA*)
226281_AT	92737	delta/notch like EGF repeat containing (*DNER*)
210814_AT	7222	transient receptor potential cation channel subfamily C member 3 (*TRPC3*)
206601_S_AT	3232	homeobox D3 (*HOXD3*)
223131_S_AT	81603	tripartite motif containing 8 (*TRIM8*)
229706_AT	10915	transcription elongation regulator 1 (*TCERG1*)
1558653_AT	339751	MAP3K20 antisense RNA 1 (*MAP3K20-AS1*)
1559826_A_AT	105379476	uncharacterized LOC105379476 (*LOC105379476*)
1553907_A_AT	161829	exonuclease 3′-5′ domain containing 1 (*EXD1*)
1569183_A_AT	1121	CHM Rab escort protein (*CHM*)
205700_AT	8630	hydroxysteroid 17-beta dehydrogenase 6 (*HSD17B6*)
1559270_AT	79776	zinc finger homeobox 4 (*ZFHX4*)
213456_AT	25928	sclerostin domain containing 1 (*SOSTDC1*)
1552280_AT	91937	T cell immunoglobulin and mucin domain containing 4 (*TIMD4*)
1565939_AT	55322	chromosome 5 open reading frame 22 (*C5orf22*)
219759_AT	64167	endoplasmic reticulum aminopeptidase 2 (*ERAP2*)
1562736_AT	56956	LIM homeobox 9 (*LHX9*)
1553513_AT	55350	vanin 3, pseudogene (*VNN3P*)
1563680_AT	284950	uncharacterized LOC284950 (*LOC284950*)
235104_AT	64167	endoplasmic reticulum aminopeptidase 2 (*ERAP2*)

**Table 4:** Top 10 hub genes.

Hub Gene	Description	Degree
*VCAM1*	Vascular cell adhesion molecule 1	472
*FHL2*	Four and a half LIM domains 2	76
*RUNX1*	RUNX family transcription factor 1	69
*TNFSF10*	TNF superfamily member 10	14
*PLAU*	Plasminogen activator, urokinase receptor	10
*SPOCK1*	sparc/osteonectin, cwcv and kazal-like domains proteoglycan (testican) 1	9
*CD74*	CD74 moleculeS	8
*SIPA1L2*	Signal induced proliferation associated 1 like 2	7
*TRIB1*	Tribbles pseudokinase 1	6
*CXCL12*	C-X-C motif chemokine ligand 12	5

**Table 5:** Details of microarray studies on sclerotic aortic valves.

Reference	Country	Dataset	Platform	Control	AS
Bosse Y. [38]	Canada	GSE83453	GPL10558	7	20
Rysa J. [14,16]	Finland	GSE51472	GPL570	10	5
Wang Y., Han D.	China	GSE155119	GPL26192	3	3
Choi B., Chang E., Song J.	South Korea	GSE77287	GPL16686	3	3

AS, aortic valve stenosis.

## Data Availability

The GEOexplorer web server is available at the following URL: https://geoexplorer.rosalind.kcl.ac.uk/ (accessed on 22 April 2023). The datasets used in the example described are available from previous studies [14,15,38], GEO Series ID: GSE83453 and GEO Series ID: GSE51472.
